# Dissecting the conformation of glycans and their interactions with proteins

**DOI:** 10.1186/s12929-020-00684-5

**Published:** 2020-09-09

**Authors:** Sheng-Hung Wang, Tsai-Jung Wu, Chien-Wei Lee, John Yu

**Affiliations:** 1 Institute of Stem Cell and Translational Cancer Research, Chang Gung Memorial Hospital at Linkou, and Chang Gung University, Taoyuan, 333 Taiwan; 2 Institute of Cellular and Organismic Biology, Academia Sinica, Taipei, Taiwan

**Keywords:** Glycan, Glycosphingolipid, Conformational changes, SPARC, Globo H Ceramide, Molecular modeling, Biacore, Molecular docking, Molecular dynamics

## Abstract

The use of in silico strategies to develop the structural basis for a rational optimization of glycan-protein interactions remains a great challenge. This problem derives, in part, from the lack of technologies to quantitatively and qualitatively assess the complex assembling between a glycan and the targeted protein molecule. Since there is an unmet need for developing new sugar-targeted therapeutics, many investigators are searching for technology platforms to elucidate various types of molecular interactions within glycan-protein complexes and aid in the development of glycan-targeted therapies. Here we discuss three important technology platforms commonly used in the assessment of the complex assembly of glycosylated biomolecules, such as glycoproteins or glycosphingolipids: Biacore analysis, molecular docking, and molecular dynamics simulations. We will also discuss the structural investigation of glycosylated biomolecules, including conformational changes of glycans and their impact on molecular interactions within the glycan-protein complex. For glycoproteins, secreted protein acidic and rich in cysteine (SPARC), which is associated with various lung disorders, such as chronic obstructive pulmonary disease (COPD) and lung cancer, will be taken as an example showing that the core fucosylation of N-glycan in SPARC regulates protein-binding affinity with extracellular matrix collagen. For glycosphingolipids (GSLs), Globo H ceramide, an important tumor-associated GSL which is being actively investigated as a target for new cancer immunotherapies, will be used to demonstrate how glycan structure plays a significant role in enhancing angiogenesis in tumor microenvironments.

## Introduction

Aberrant expression of sugar is a unique feature of cancer cells [8, 18, 25, 29, 73]. In this rapidly advancing era of anticancer therapeutics, most of the available therapeutics are directed against proteins/glycoproteins. The profile of glycans expressed on cell surface are correlated with cell types; for example, specific sugar signatures of cancer cells were observed [18, 72, 73]. Therefore, glycans are used as targets of therapeutic development because their expressions and the conformations required for molecular recognition in cancer are usually unique [18, 72, 73]. Compared with traditional small molecule anticancer drugs, the structural basis for therapeutic design in the targeting of sugar-protein complexes for clinical use has not been widely investigated. On the other hand, computer-aided methods play a useful role in the rational design of therapeutics targeting protein molecules [56, 59]. In this review, the development and application of in silico strategies for investigating the molecular interactions between glycans and proteins will be highlighted.

Core fucosylation is an important post-translational modification of the N-glycan core structure, forming the α1,6 fucosylation of the N-acetylglucosamine (GlcNAc) residue linked to the asparagine, catalyzed by fucosyltransferase 8 (Fut8). Fut8 has little homology with other fucosyltransferase (Fut) members and most Fut8 knockout mice die shortly after birth, accompanied with multiple organ dysfunctions [63], suggesting its important roles in many biological functions. It has also been reported that core fucosylation modified by Fut8 seems to be crucial for the ligand binding affinity of TGF-β1 receptor, EGF receptor, and integrin α3β1-mediated signaling and biological function [60–63]. Dr. N. Taniguchi further reported that Fut8^−/−^ mice develop an emphysematous phenotype in the lung [63]. In addition, a decrease in Fut8 activity is observed in cigarette smoke-exposed mice, which is related to the development of chronic obstructive pulmonary disease (COPD) [15]. Furthermore, matrix metalloproteinases (MMPs) have been implicated in the pathogenesis of COPD, and increased expression of these proteinases has been reported in lungs of Fut8^−/−^ mice [63]. In spite of these studies in Fut8^−/−^ mice, the precise roles of *Fut8* in alveologenesis during lung development and the formation of emphysema in lung diseases have not been investigated in detail either *at cellular or molecular levels*.

In our recent studies, to investigate how the posttranslational modulation by which changes of core fucosylation affects the functions of lung, we utilized the quantitative mass spectrometry (MS) proteomic technology platform to explore the site-specific changes of core fucosylation for Fut8 targeted proteins. While MS proteomic analysis had revealed many candidate proteins as core fucosylated targets of Fut8, we were interested particularly in secreted protein acidic and rich in cysteine (SPARC). SPARC belongs to the matricellular family member that modulates interactions between cells and extracellular matrix (ECM) in many physiological processes, including cell adhesion, cell turnover and embryonic development [4, 70]. SPARC binds multiple structural and soluble ECM proteins in a calcium-dependent manner. The binding of SPARC to collagens requires non-denatured, triple helical collagen [22]. Expression of SPARC was reported to be increased in adult tissue during inflammation, tissue injury and remodeling, suggesting its importance in tissue regeneration and repair [2, 65]. In solid tumors, the ECM, which serves not only as the scaffold for the tumor to grow but also provides critical regulatory functions to sustain the progression and modulate hallmarks of cancer, is consisted of a meshwork of structural molecules (such as collagens and fibronectin) and the non-structural regulatory, matricellular proteins like SPARC family proteins [39]. These matricellular family proteins include SPARC, OPN (Spp1), tenascin, TSPs, POSTN, and the CCN family [39]. SPARC has been chosen for discussion in this review because it was shown to act as a collagen chaperon and regulate the binding for collagen structural proteins in the ECM. It may thus sequester and modulate the activities of specific growth factors, involving in functions in physiologic tissue repair and pathologic conditions of tissue remodeling, such as fibrosis and tumorigenesis [10, 43, 50].

In addition, it was shown that during tumorigenesis, high SPARC expression in tumor cells promotes epithelial mesenchymal transition (EMT) and tumor aggressiveness [44]. Different immune cell types including regulatory T cells, myeloid-derived suppressor cells (MDSCs), and tumor-associated macrophages were reported to mediate immunosuppression in the tumor microenvironment. Recent studies demonstrated that an aberrant ECM deposition as characterized by SPARC and high collagen content promotes the recruitment of suppressive myeloid cells [44]. Therefore, SPARC may also play an important role in regulation of the interaction between collagen deposition and leukocyte recruitment to drive the induction of EMT in tumor microenvironment [28, 42].

COPD is a progressive disease with high morbidity and mortality worldwide. Various studies have shown increased degradation of collagens in the matrix of lungs indicating that ECM turnover is important in the pathology COPD [42, 48]. However, the role of SPARC-collagen interactions associated with the pathogenesis of COPD remain poorly understood. Since SPARC regulates crosstalk between cells and collagen, an understanding how the glycosylation affects the interactions between SPARC and collagen will be important.

Additionally, we discuss another class of glycomolecules, glycosphingolipids (GSLs) and their interactions with proteins. GSLs, which contain a ceramide with attached carbohydrates, are a subclass of glycolipids found in all eukaryotic plasma membranes and display diverse functions in normal and cancer cells [18]. Recent FDA approval of anti-GD2 (a ganglioside) monoclonal antibody (dinutuximab) for neuroblastoma marked the first *non-protein* GSL to be used as a target for anticancer therapy [72]. We previously reported that there were unique dynamic changes in GSL composition during human embryogenesis [16, 18, 73]; for example, Globo H ceramide (GHCer), which consists of a hexasaccharide linked to a ceramide, Fucα1-2Galβ1–3GalNAcβ1–3Galα1-4Galβ1-4Glcβ1Cer, is abundantly expressed in undifferentiated embryonic stem cells (ESCs) and disappears after differentiation [16, 18, 73]. But importantly, GHCer is the most prevalent cancer-associated GSL. Currently, GHCer is being actively pursued as the next non-protein antigen target for cancer immunotherapy [12, 19], because it is overexpressed in many epithelial cancers [8, 18, 73]. A multinational phase II randomized clinical trial of Globo H vaccine has shown greater progression-free survival of breast cancer patients who developed significant anti-Globo H antibodies than a placebo group [19] . In addition, a global phase III randomized clinical trial is ongoing. However, the biological functions of GSLs remain underexplored. In this review, we will highlight the features of molecular interactions between GSL and proteins, especially how conformational changes of glycans in GSL could lead to alterations in protein interactions and their biological consequences. In addition, we will discuss the roles of fucose in producing several biological consequences after affecting conformational changes in glycans to alter their interactions with proteins in cells. Thus, we will provide scientific rationales for targeting GHCer in anticancer therapy and elucidate the structural basis for a rational design of GHCer-targeted therapeutics.

## Main text

### Technology platforms for assessment of glycan-protein interactions

The use of in silico strategies to develop the structural basis for a rational design of glycan-targeted therapeutics remains a great challenge. This problem derives, in part, from the lack of technologies to quantitatively and qualitatively assess the complex assembly between a glycan and the targeted protein molecule. For example, difficulties with the analysis of glycan-protein complex assembly arise from the exceptional flexibility of sugar moieties in solution [35, 66], various forms of glycan conformers having different energy states [35, 66], and many different molecular interactions within the complex. All of these variations make the assessment and optimization of glycan-protein complexes highly time consuming. In addressing the unmet need for developing new sugar-targeted therapeutics, many investigators are searching for technology platforms suitable for elucidating various types of interactions within glycan-protein complexes. Below we discuss three important technology platforms commonly used for quantitative and qualitative assessment of complex assembly.

#### Biacore analysis

**Fig. 1:**
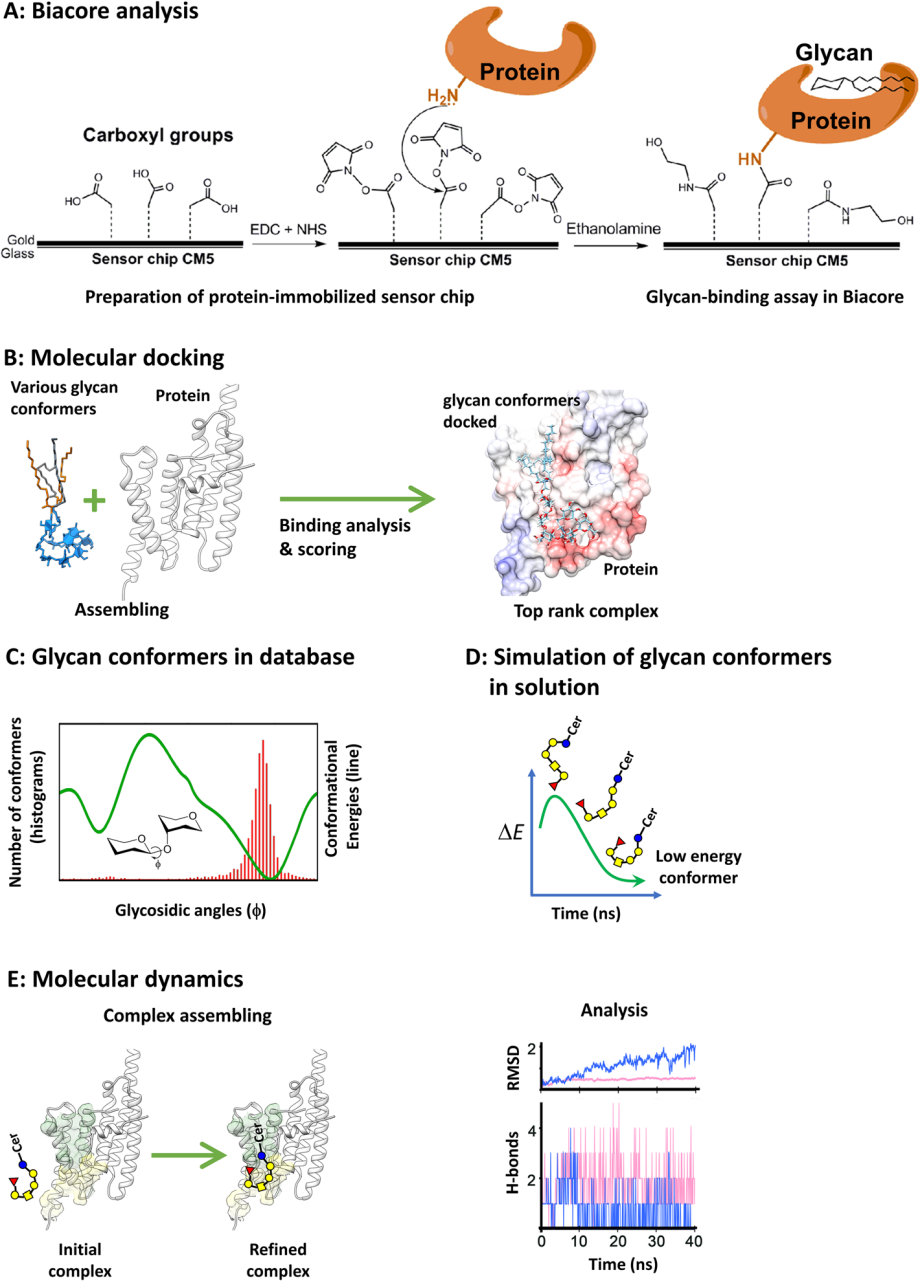
Biacore analysis, molecular docking and molecular dynamics. **a** For analysis of protein-glycan interactions using Biacore, recombinant protein is immobilized on the sensor chip. Generally, the carboxyl groups on CM5 chips, which are made of carboxymethyl-dextran matrix, are activated by EDC (1-ethyl-3-[3-dimethylaminopropyl]-carbodiimide hydrochloride) and NHS (N-hydroxysuccinimide). Subsequently, the primary amines on the protein form amide linkages with these activated carboxyl groups, followed by deactivation using ethanolamine. Therefore, the Biacore system can detect the association and dissociation of glycan molecules that interact with the proteins on the sensor chip. **b** Molecular docking programs can randomly generate conformers of glycans and proteins, and this is followed by assembly and scoring of their complex structures. Binding analysis for the top rank complexes, which are predicted by good scores, is useful for prediction of glycan-binding sites with various types of molecular interactions. **c** The conformational energies of glycan (solid line) vary with the glycosidic angles. The conformers of glycans (histograms) found within database of protein-glycan complexes are mainly distributed at the low energy states. **d** Molecular dynamics software can generate low energy conformers of a glycan molecule by simulation of its molecular motion in solution. **e** The low energy glycan conformer is then used to simulate the formation of the protein-glycan complex. The molecular interactions and stabilities of these complexes can thus be evaluated by methods such as calculation of RMSD and H-bonds

Biacore is an assay for the measurement of biomolecular complexes such as small compound- or protein-protein complexes, which does not require the use of fluorescent or radioisotope labeling. The optical detection in a Biacore system is based on surface plasmon resonance (SPR) technology: the SPR signals, which are displayed as response units (RU) in the sensorgrams, correlate with the mass of biomolecules bound to the surface of a sensor chip (Fig. 1a) [56, 58]. Conventionally, for analysis of glycan-protein interactions using Biacore, recombinant protein is immobilized on the sensor chip through the formation of amide linkages between the protein and the carboxymethyl-dextran matrix of the CM5 chip (Fig. 1a) [56, 58]. Then, Biacore estimates the binding kinetics and affinities by monitoring the association and dissociation of the glycan molecule (Fig. 1a) with the attached protein in real time [56, 58]. Subsequently, the rates of association and dissociation of the complex on the chip are evaluated at various concentrations of glycan [56, 58]. The concentration-dependent curves are then used to estimate the dissociation constant (Kd). On the other hand, glycans might be captured by antibodies on the chip followed by analysis of its binding with various concentrations of the protein in solution [11]. However, since antibodies on the chip might hinder the interactions between the glycan and its interacting protein, this capturing method may not suitable for all types of glycans. Labeling molecules with linking tags, such as biotin, which can be easily captured by streptavidin on sensor chip, has been applied for binding assay using Biacore [11].

#### Molecular docking

Determination of the complex structures of glycosylated biomolecules or glycan-protein complexes using traditional methods such as X-ray crystallography and NMR spectroscopy involves many challenges. The difficulties involve, for example, the production and purification of the protein, the synthesis and isolation of the glycan or glycoconjugates, and the co-crystallization of the glycan-protein complex [67]. But, there is long-standing interest in using computer-aided methods for the characterization of complex structures containing glycans or glycosylated biomolecules [35] and, especially, for the rational design of interactions between proteins and their binding partners [56, 57, 59]. Molecular docking is an in silico modelling method which involves the interaction between two or more component molecules to form stable complexes [56, 57, 59]. For molecular docking studies, the conformers of glycan and protein are calculated, individually, followed by the assembly and scoring of the complex structures (Fig. 1b), using one or more scoring algorithms to rank the complex according to binding scores (Fig. 1b) [57]. Scoring programs, such as HotLig, calculate parameters for various types of molecular interactions, including H-bonds, ionic interactions, hydrophobic effects, and many other factors affecting molecular binding in complex assembling [57]. Depending upon the analysis of these parameters, molecular docking predicts the binding modes in complex structures with success rates of about 80–90% [57]. In which studies, the criteria for success prediction of binding modes via scoring of various molecular interactions, were referred to that the root-mean-square deviation (RMSD) values between predicted and experimental coordinates of binding ligands were smaller than 2 Å [57]. For rational drug design and discovery, molecular docking often generates a reasonably attractive complex for elucidating structure-activity relationships [56, 57]. Therefore, molecular docking is commonly used to conduct virtual screening of drug candidates [56, 57]. Additionally, molecular docking is useful for the prediction of binding sites involved in molecular interactions [56, 57].

#### Molecular dynamics

However, a glycan can be a compound or the carbohydrate part of a glycoconjugate, consisting of a number of monosaccharides linked glycosidically. These glycosidic linkages are formed at multiple potential sites in saccharide molecules, and glycans can have complicated structures with various branched configurations [20, 21]. Since glycans are not only complicated in composition but also vary in conformations, prediction of glycan-protein complex assembly using molecular docking often encounters difficulties, resulting in a reduction of prediction accuracy and time-consuming calculations. In a detailed analysis of glycan conformers using various experimental complexes (Fig. 1c), it was found that glycan conformers in the complex assembly (red histogram) possessed glycosidic angles (ϕ) distributing mostly in the region where the conformers were at their low energy states (solid green line) [35, 66]. On the other hand, the probabilities of finding glycan conformers present in glycan-protein complexes decreased remarkably with the conformers possessing higher conformational energies (Fig. 1c) [35, 66]. In other words, low-energy glycan conformers would be more likely to form stable complexes with protein molecules, as compared to glycan conformers with higher energy states. These results suggest that the selection of conformers with low-energy states determined from the energy function of glycosidic angles for molecular docking could improve predictive power and accuracy in the assessment of stable glycan-protein complexes [35]. Out of these considerations, molecular dynamics could be a useful computational method, particularly for the analysis of glycan-protein complexes, because this approach employs not only modeling of the 3D structure of the complex, but also analysis and refinement of various spatial restraints for assembly.

Algorithms for such molecular dynamics simulations have been well developed; software packages for molecular dynamics simulations include GROMACS [40], NAMD [38], AMBER [7] and CHARMM [5]. Based on the atomic coordinates at each timestep during dynamics simulation, the trajectories of molecular motion have been used to survey the conformers of component molecules [5, 7, 38, 40]. Moreover, molecular dynamics simulations have been used for optimization of the conformers, as well as the evaluation of structural stabilities [5, 7, 38, 40]. Performing molecular dynamics simulations with glycan-protein complexes requires the generation of molecular topologies and parameters with appropriate force fields for the component molecules, such as atom types, hybrid orbitals, and charges [13, 26]. In silico tools, such as doGlycans [13] and Glycosylator [26], have been used to generate the structural coordinates and topologies of glycans. The software programs doGlycans and Glycosylator can both deal with glycoproteins, while doGlycans is also applicable for glycolipids and glycan polymers [13].

The strategy of using molecular dynamics simulations to build models of glycan-protein complexes and analyze the molecular interactions within the complexes is illustrated in Fig. 1d-e. In order to avoid the generation of an unstable complex assembly, low-energy glycan conformers (Fig. 1d) must be found. For example, with GSLs in solution, molecular dynamics simulations could reveal various glycan conformations having different levels of energy (Fig. 1d). Unstable glycan conformers with high-energy are prone to convert to lower energy states, when approaching a dynamic equilibrium (Fig. 1d) [21]. Through enumeration of conformational energies (kcal/mol), the low energy conformers of glycans can be optimized and identified (Fig. 1d) [21]. Then, low energy conformers can be assembled into complexes with the targeted protein using software such as GROMACS in another molecular dynamics simulation (Fig. 1e). To avoid time-consuming calculations, the low energy glycan conformer can be manually placed in proximity of the binding site of the protein molecule (Fig. 1e). Afterwards, the molecular interactions among various components of this initial complex can be optimized using molecular dynamics simulation for refinement of various physical restraints for complex assembly (Fig. 1e). While analyzing the molecular dynamics data, the motions of the component molecules in the simulation should reach a dynamic equilibrium in which the conformers no longer change their molecular interactions within the complex. As shown in Fig. 1e, when complex assembly reached a state of dynamic equilibrium, the “root-mean-square deviation” (RMSD) values became stabilized after a period of simulation (red line). In contrast, if a stable equilibrium state was not obtained, the RMSD values increased continually with time (blue line in Fig.1e). In addition to conformational changes, the association or dissociation among various components in the glycan-protein complex can also be assessed. As a result, the interactions either inter- or intra-molecularly within the complex could be revealed. For example, the number of actually occurring H-bondings could be revealed, as shown in Fig. 1e.

### How conformational changes of glycans influence SPARC-collagen interactions

Glycosylation is the most common post-translational modification. The carbohydrate structures of surface glycoconjugates play an important role in many physiological and pathological events [36, 41]. Fucosylation is a post-translational modification, involving the addition of fucose from GDP-fucose to glycoconjugates, in reactions catalyzed by Fut. To date, 13 Futs have been identified in the human genome [14, 23, 47, 52]. Fut8 is the only Fut responsible for the important modification of *N*-glycans, involving α1, 6 fucosylation of the GlcNAc residue linked to the asparagine of glycoconjugates (core fucosylation) [32, 53]. Fut8 has little homology with other Fut members and plays essential roles in biological functions as evidenced by the report that 70% of Fut8-null mice died shortly after birth [63]. The level of core fucosylation has also been implicated in the development of emphysema in Fut8^−/−^ mice and cigarette smoke-exposured Fut8^+/−^ mice [15, 63]. In addition, Fut8 had been shown to have a significant impact on cell growth and differentiation [62]. We also found that core fucosylation in lung stem/progenitor cells was altered by chemicals or environmental risk factors, such as polycyclic aromatic hydrocarbons, leading to lung disorders (Tseng YH et al.: Benzo[α]pyrene induces fibrotic changes and impairs differentiation for lung stem cells, submitted). However, the precise biological roles of Fut8 have not been investigated in detail at either the cellular or molecular level. Therefore, in our studies, we have employed a quantitative site-specific MS proteomic platform to analyze the core fucosylated proteins after Fut8-knockdown (Fut8-KD) of lung stem cells [68]. We employed the following two criteria to gauge the relative contributions of core fucosylation to posttranslational modification of target proteins: i) a significant decline in core fucosylated peptides based on the MS quantitative analysis and ii) lack of changes of gene expression in the cDNA microarray after Fut8-KD. While MS proteomic analysis had revealed many candidate proteins as core fucosylated targets of Fut8, we were interested particularly in SPARC, which modulates interactions between cells and extracellular matrix [4].

#### Glycan compositions vs SPARC-collagen interaction

**Fig. 2:**
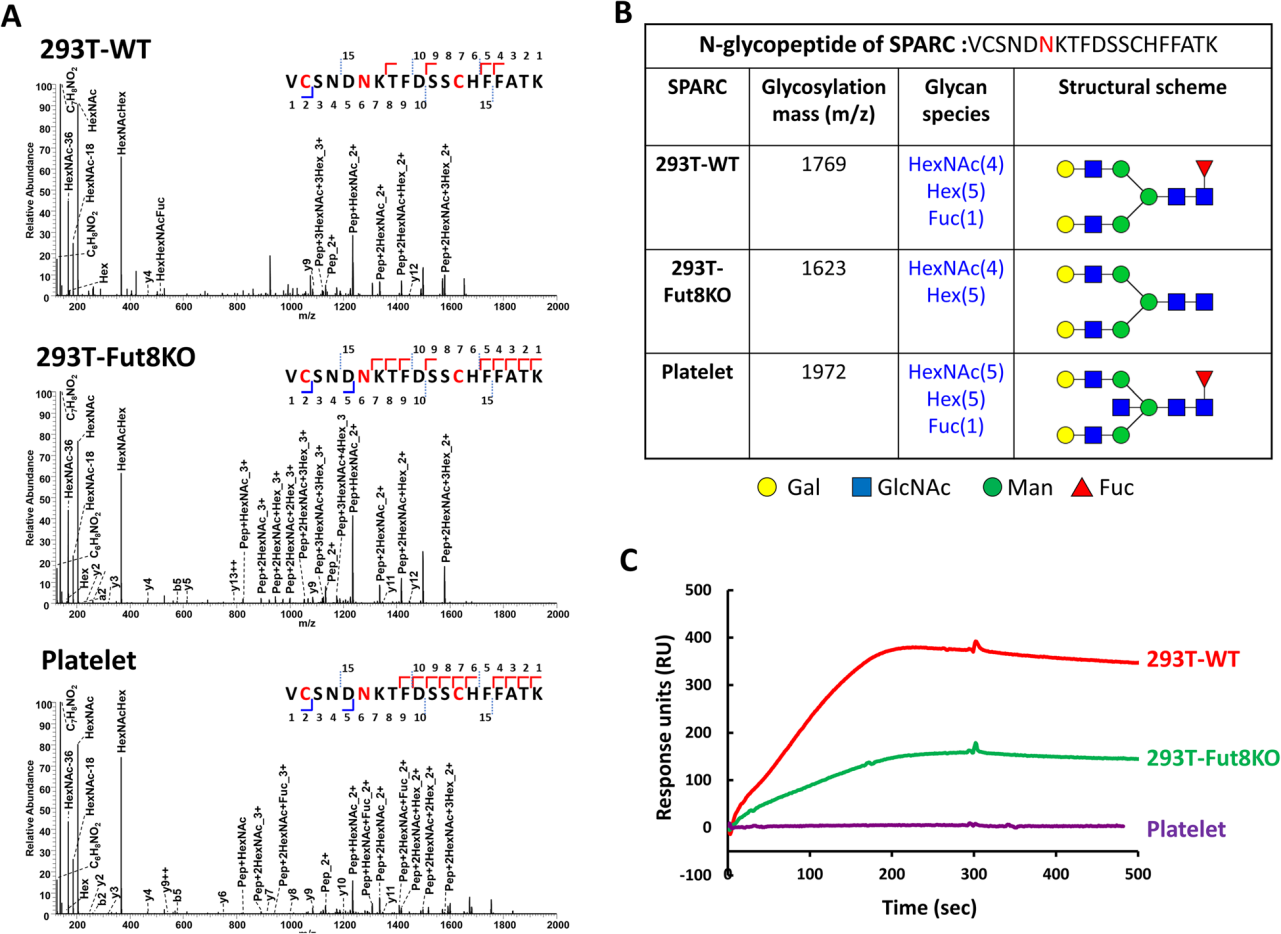
Effects of glycan compositions of SPARC from different sources on binding to collagen I. Analyses of the N-glycopeptide VCSNDN^116^CFKTFDSSCHFFATK (AA 111–129) of SPARCs from 293 T-WT cells, 293 T-Fut8KO cells and platelets were performed using LC-MS/MS with electron-transfer/higher-energy collision dissociation (EThCD) techniques and Byonic software. EThCD-MS/MS spectra assignments for the b and y fragment ions from the peptide backbone were observed and annotated, which could be used for the sequencing of the peptide. Peptide sequences are depicted in the top right corners. The dissociation with high energy resulted in specific glycan oxonium ions derived from the fragmentation of the N-linked GlcNAc. The summary of peak assignments was shown. **a** Information for each glycoform in N116 of SPARC, including the m/z of glycosylation, glycan species, and structural composition are depicted. **b** Biacore analysis of the binding of SPARC from different sources to collagen I. SPARC (1 μM) from 293 T-WT cells (red), 293 T-Fut8KO cells (green), and human platelets (purple), was injected onto separate collagen I-coated sensor chips

SPARC is a matricellular protein that regulate cell-matrix interactions and cell function. Different from traditional matrix proteins, such as fibronectin, collagen and laminin, which contributed to the structural stability of the ECM, matricellular proteins have been classified as a non-structural regulatory matrix proteins capable of modulating a variety of biological processes for the ECM [3]. Expression of SPARC is elevated during embryogenesis and is diminished in normal adult tissue. In addition, its expression appears to correlate with tissue injury, regeneration, inflammation, and malignant tumor growth. Although SPARC is reported to bind a number of ECM structural proteins, SPARC binding to collagens is the best characterized of these interactions. Previous studies have showed that SPARC binds to fibril-forming collagen types I, III, and V, and the basement membrane collagen type IV [46]. Limited cleavage of SPARC by matrix MMPs increases its affinity for collagens [45]. In addition, tissue-specific differential glycosylation of SPARC also influences SPARC binding to collagen [22]. SPARC is a N-linked glycoprotein shown to undergo differential glycosylation in a tissue-specific manner [22]. It has been reported that bone SPARC carries high mannose-type glycans and platelet SPARC has complex-type glycans. The difference in glycosylation appeared to have functional consequences, such as the bone SPARC bound to collagen I, III, and V in solid phase assays, whereas the platelet SPARC did not [24]. However, the glycan role in the conformational dynamics of SPARC and in the presence of the collagen complex was not investigated. In addition, to study the effect of core fucosylation on SPARC, full-length human SPARC was expressed and purified from transfected control 293 T-WT (wild type) and 293 T-Fut8KO (knockout) cells. We employed liquid chromatography-tandem mass spectrometry (LC-MS/MS) analysis with electron-transfer/higher-energy collision dissociation (EThCD) techniques and Byonic software to examine the N-glycan structures attached to N116 of SPARC proteins from different preparations, including the recombinant SPARC proteins from 293 T-WT and 293 T-Fut8KO cells, and the SPARC proteins obtained commercially from human platelets (Fig. 2a). The major composition and structural differences between N-glycans attached to the glycopeptide VCSNDN^116^CFKTFDSSCHFFATK at N116 are shown in Fig. 2b. Core fucosylation at N116 via GlcNAc was confirmed in the SPARC from 293 T-WT cells, but not 293 T-Fut8KO cells. In addition, the N-glycan structures attached to this glycopeptide of SPARC from both 293 T-WT and 293 T-Fut8KO cells were the biantennary complex type oligosaccharides with or without core fucosylation, respectively (Fig. 2b). On the other hand, the SPARC obtained from platelets was mainly of the complex type, containing a core fucose and a bisecting GlcNAc moiety (Fig. 2b).

Subsequently, these purified SPARC proteins were examined using the Biacore assay for their binding to collagen. As shown in Fig. 2c, SPARC from 293 T-WT cells showed the highest affinity of binding with collagen I. In contrast, collagen binding by SPARC from 293 T-Fut8KO cells was markedly decreased. Moreover, the SPARC proteins derived from platelets did not bind to the collagen-coupled chips used in the Biacore assay (Fig. 2c). These results are consistent with previous analyses [22, 24], which showed that bone-derived SPARC (complex type/high mannose) binds collagen with higher affinity than platelet-derived SPARC. Overall, these results showed that there are different glycan compositions in tissues from different sources, which exhibit variations in affinities for collagen binding.

#### Structural modeling for SPARC protein containing complex type N-glycans

**Fig. 3:**
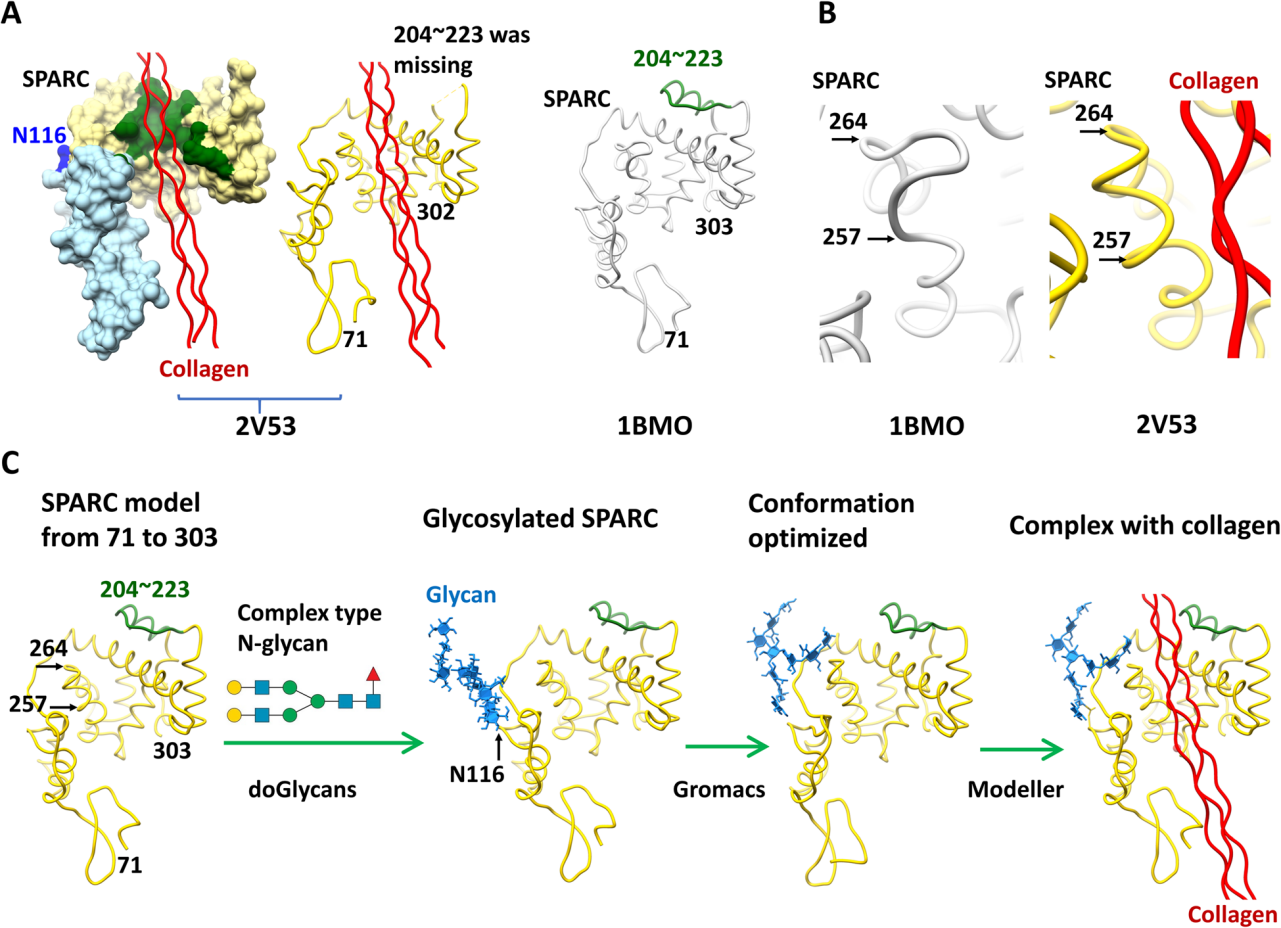
Modeling of structures and molecular interactions for the complex between N-glycosylated SPARC and collagen. **a** The Fut8 target protein, SPARC, is a collagen-binding matricellular protein. Surface representation of the crystal structure of human SPARC includes the follistatin-like (FS) domain (cyan) and the C-terminal extracellular calcium-binding (EC) domain (yellow) bound to collagen peptide (red) [Protein Data Bank (PDB; http://www.rcsb.org), code 2 V53]. N-glycosylation at site N116 of SPARC is highlighted in blue, and collagen binding residues of SPARC are highlighted in green. Another structural model of SPARC protein, 1BMO, contains the peptide fragment AA 204 to 223, which was lacking in the model of PDB 2 V53. **b** The 1BMO model did not form a complex with collagen. Its AA 257 to 264 sequence displayed a unique loop conformation. When SPARC complexed with collagen in 2 V53, the AA 257 to 264 segment was folded to form a helix structure. **c** Modeller software was used to generate a new SPARC model, using 2 V53 and 1BMO as templates. The complex type N-glycan moiety was then attached to the side chain of N116 of SPARC using doGlycans software. The conformation of glycosylated SPARC protein was then optimized using molecular dynamics simulation software, e. g., Gromacs. Finally, Modeller software was used to model the molecular interactions between the glycosylated SPARC and collagen molecule

Since the glycan structures of SPARC proteins varied with different tissues, presenting different binding affinities for collagens [22], it would be of interest to examine how the composition of different N-glycans in SPARC affects the molecular interactions with collagen. The human SPARC protein is composed of 303 amino acids (AA), including the AA 1 to 17 signaling peptide, which was removed during subsequent protein processing [54]. In this review, the numbering of AA sequence started with the first AA of the N-terminal signaling peptide of SPARC (UniProtKB ID: P09486). SPARC is an evolutionarily conserved glycoprotein, consisting of three protein domains: a flexible N-terminal domain, a follistatin-like (FS) domain, and a C-terminal extracellular calcium-binding (EC) domain [4, 30]. To delineate an *in silicon* structural 3D model for SPARC, we first searched the Protein Data Bank (PDB) (http://www.rcsb.org) and found two tentative structural models (PDB: 2 V53 and 1BMO) for fragments of human SPARC, each consisting of FS and EC domains (Fig. 3a).

As shown in Fig. 3a, the 2 V53 model represents the X-ray crystallographic structure of a SPARC-collagen complex containing the AA sequence 71 to 302 (with the FS and EC domains shown in blue and yellow, respectively, in the surface model) and the triplex-helix collagen (red). In the modeling, if the separation between carbon atoms of SPARC and collagen was less than 5 Å in distance, the area covered by such close contact was considered to be a binding site for two proteins in the complex (green in Fig. 3a). Furthermore, it is noted that there was only one N-glycosylation site for the SPARC molecule, which was reported to be at N116 in the FS domain [22, 69] (Fig. 3a).

The 2 V53 model did not provide structural information for the AA 204 to 223 segment (ribbon model in Fig. 3a). The other structural model of SPARC protein, 1BMO (Fig. 3a), also based on crystallographic data, contains similar FS and EC domains (AA 71 to 303 segment). However, 1BMO model of SPARC was built, not to be in complex with collagen structure; but this data set contained the atomic coordinates for the peptide fragment with AA 204 to 223, which was lacking in the 2 V53 model. These two structural models of the SPARC protein also displayed different conformations in the region AA 257 to 264 (Fig. 3b). For the SPARC protein in 1BMO, which did not form a complex with collagen, the AA 257 to 264 segment displayed a unique loop conformation (Fig. 3b), effectively shielding against collagen binding. In comparison, when SPARC complexed with collagen, the AA 257 to 264 segment of SPARC in 2 V53 was notably folded to form a helical structure (Fig. 3b), thus enabling the access of collagen to its binding site. Overall, for apo form of SPARC, the AA 254 to 264 segment shielded collagen-binding site. In contrast, the conformation of this AA 254 to 264 segment can change to the collagen-binding form, resulting in the opening of the binding site. In conclusion, the conformation of the AA 257 to 264 segment of SPARC seemed to be an important element involved in the binding with collagen (Fig. 3b).

In order to study the conformational changes of this part of the human SPARC protein, we generated a protein model of the SPARC molecule that contains not only complete AA sequences, but also exhibits a suitable conformation for collagen binding. This model employed the strategies of both homology modeling and molecular dynamics simulation to construct the 3D structure of SPARC containing its glycan moiety and retaining the ability to bind collagen (Fig. 3c). The impact of compositional differences in the glycan moiety on the molecular interactions of SPARC with collagen was also investigated.

Homology modeling is a method for prediction of 3D structures of target AA sequences based on the known structures of templates with homologous sequences [33, 55]. Examples of software programs for homology modeling include Modeller [64], Nest [37], and SegMod/ENCAD [27]. First, after searching for template structures in the PDB, homology modeling programs can designate parts of known, but related 3D structures as templates for sequence alignments with the target sequences. After the target–template sequence alignment is established, the next step in homology modeling is model building with the Modeller program. Next, spatial restraints, dihedral angles, and other physical parameters of the 3D protein structure of the target sequence could be calculated from alignment with template structures. Finally, the protein model could be refined with gap deletion, loop addition, sugar conjugation, and energy-optimization of the main chain and side chains of the structure [33, 55].

As illustrated in Fig. 3c, for homology modeling of human SPARC sequences, we used the Modeller software to generate a new SPARC model with the peptide domain AA 71 to 303, using the 2 V53 and 1BMO models from PDB as templates. The helical conformation of the AA 257 to 264 segment for collagen binding was adopted using 2 V53 as a template. On the other hand, the missing information from AA 204 to 223 in 2 V53 was built into the model, using the information from the corresponding peptide fragment in 1BMO. Next, to evaluate the role of the conformation of N-glycans in collagen binding, complex type N-glycans, for example, biantennary or bisecting N-glycans (Fig. 2b), were added into the model using doGlycans software (Fig. 3c). For modelling of the glycan conformations, various connections and configurations of saccharide units of the glycan were described by a series of specific codes, as defined in doGlycans. These codes were then designated with d-, l-, α-, and β-forms for the carbohydrate chains; their glycosidic angles were also definable parameters for input. Through calculation of spatial restraints, the complex type N-glycan moiety was attached via GlcNAc covalently to the side chain of N116 of SPARC. Afterwards, modeling of the glycosylated SPARC protein was subjected to energy optimization for conformations using molecular dynamics simulations software, e. g., Gromacs (Fig. 3c). On reaching equilibration, the detailed intramolecular interactions between the N-glycan and SPARC could be revealed. Finally, Modeller software was used to model the complex assembly between the glycosylated SPARC and collagen molecule (Fig. 3c). Thus, this model of SPARC protein from AA 71 to 303 (Fig. 3)c possesses two structural features for the complex: (i) core fucosylation at N116 of SPARC via GlcNAc, and (ii) helical conformation of the AA 257 to 264 segment of SPARC for collagen binding.

#### How the glycan structure affects the SPARC-collagen interaction

The only N-glycosylation site in human SPARC is located at N116 [69], where core fucosylation takes place (Fig. 3a). As shown, this N116 is far away from the site of collagen binding by SPARC (green area in Fig. 3a). Therefore, it would be interesting to examine how the N-glycan structure attached at N116 affects the complex assembly between SPARC and collagen.

**Fig. 4:**
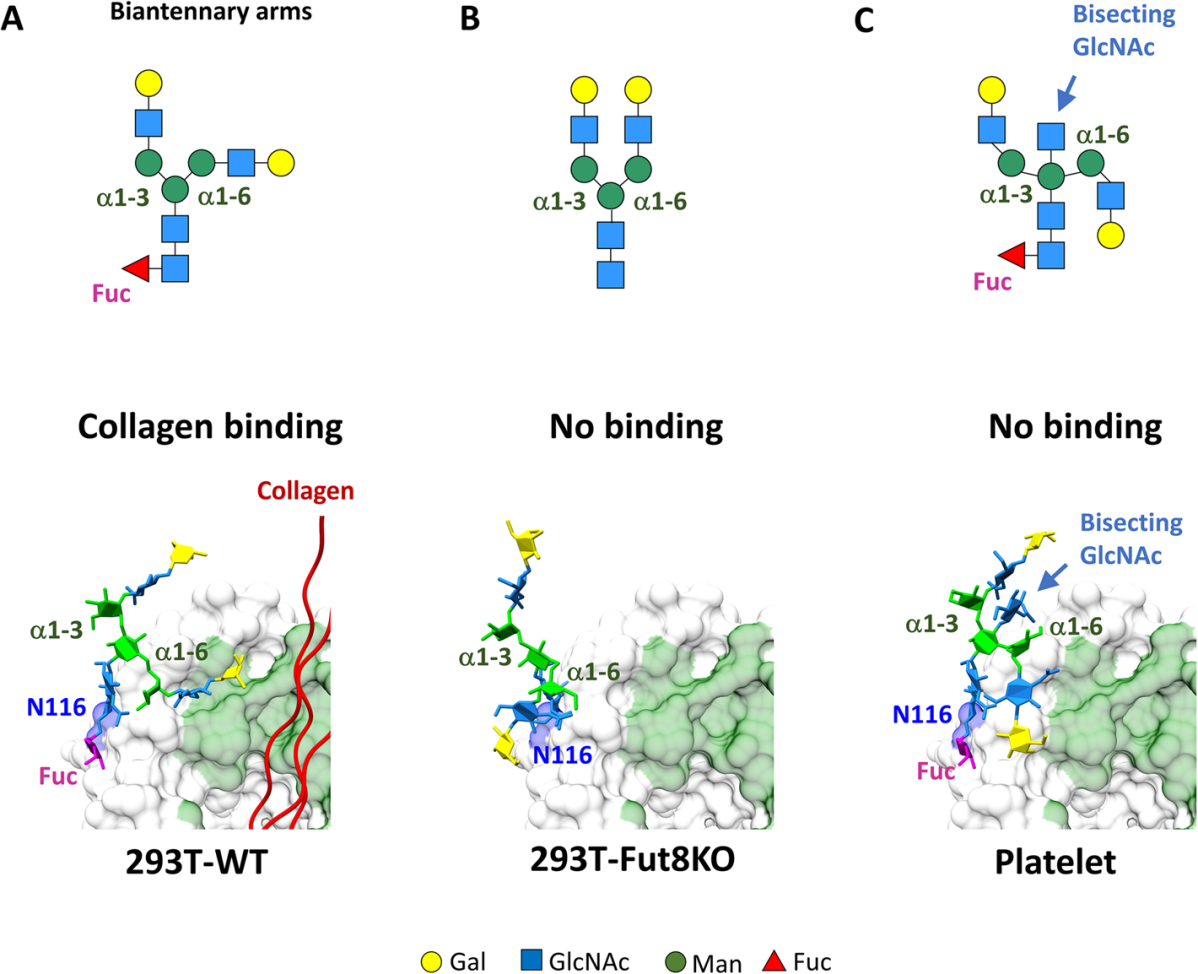
Molecular dynamics simulation revealed that the conformation of glycan regulated the binding between SPARC and collagen. Comparison of the conformation of the biantennary or bisecting N-glycans of SPARC from 293 T-WT cells (**a**), 293 T-Fut8KO cells (**b**), and platelets (**c**) by molecular dynamics simulation (lower panels). The upper panels show schematic drawings of the conformational changes of the N-glycans of SPARC

As previously described, molecular dynamics simulations are useful for the identification of conformational changes and evaluation of molecular interactions within the complex assembly. Since different N-glycans were found in SPARC proteins obtained from 293 T-WT cells, 293 T-Fut8KO cells and platelets (Fig. 2b), we generated 3D structural models of SPARC containing various complex-type N-glycans. Similar to the approaches used in Fig. 3c, doGlycans software was used to generate 3D structures with three different types of glycans (Fig. 2b), using 2 V53 as a template, which in turn was simulated using GROMACS [13, 17] and further energy-optimized. After a period of 20-ns simulations models of N-glycosylated SPARC protein with different N-glycans for 293 T-WT cells, 293 T-Fut8KO cells, and platelets were built with or without collagen binding (bottom panel in Fig. 4).

To illustrate the structural impact of N-glycans, we examined the molecular dynamics simulation results for SPARC derived from 293 T-WT and 293 T-Fut8KO cells (Fig. 4a and b). With the core fucosylated GlcNAc covalently linked to N116 in the SPARC protein, the conformer of this complex type N-glycan obtained from 293 T-WT cells (Fig. 4a) showed a configuration with one biantennary arm, which was on the Manα1–6Man linkage, bending over to reach the collagen binding site of SPARC (green surface in lower panel of Fig. 4a). It was further shown that as this biantennary arm extended toward the collagen binding site, its terminal galactose and GlcNAc residues formed hydrogen bonds and hydrophobic interactions with SPARC protein at K150, P261 and H264 (Fig. 4a). In other words, in 293 T-WT cells, such close interactions of its N-glycan with the SPARC protein moiety might regulate the protein conformation of SPARC, facilitating its binding to collagen. Notably, the P261 and H264 of SPARC, which were found to interact with the biantennary arm of the N-glycan, were located within the AA 257 to 264 segment of SPARC (Fig. 3b), which created a unique open conformation in the 2 V53 model to facilitate the access of collagen to its binding site with SPARC.

In contrast, for the N-glycan obtained from 293 T-Fut8KO cells, which lacked core fucosylation, molecular dynamics simulation showed that both biantennary arms of the N-glycan attached at N116 were freely extended with full flexibility and that neither arm made contact with the SPARC protein or collagen molecule to enable interactions (Fig. 4b). Therefore, when core fucosylation occurred with the GlcNAc attached to N116 of the SPARC in 293 T-WT cells, the glycan conformer altered the conformation of SPARC allowing it to maintain a helical configuration at AA 257 to 264, and thereby exhibit high affinity binding to collagen (2 V53 in Fig. 3b). On the other hand, with the absence of core fucosylation, the AA 257 to 264 segment of SPARC might acquire a loop conformation (1BMO in Fig. 3b), resulting in lower affinity binding with collagen. In other words, the core fucosylated glycan may serve to stabilize the SPARC structure by causing a conformational change that facilitates SPARC binding to collagen.

Therefore, core fucosylation of the N-glycan in SPARC affects the conformation of the complex type of glycan and its interactions with SPARC protein, which in turn affects access to the collagen binding site of SPARC. Furthermore, the bisecting GlcNAc with core fucosylationin SPARC from platelets displayed a totally different picture of glycan in molecular dynamics simulations, as shown in Fig. 4c. It was found that the atomic motion of the two biantennary arms of the N-glycan was restrained to become less flexible due to steric hindrance caused by the existence of bisecting GlcNAc in the molecule (Fig. 4c), resulting in a unique configuration of both N-glycan arms, which could not make contact with the AA 257 to 264 segment to produce a conformational change and enable collagen binding. In conclusion, the arm on the Manα1–6Man linkage was more flexible than the arm on the Manα1–3Man linkage (Fig. 4), because the α1–6 linkage possessed three glycosidic bonds, whereas the α1–3 linkage contained only two glycosidic bonds. The core fucosylation promoted the orientation of the biantennary arms toward the collagen-binding site on SPARC, especially the arm on Manα1–6Man linkage (Fig. 4a). However, the bisecting GlcNAc played a critical role in restricting the flexibility of both biantennary arms, resulting in low affinity binding of SPARC to collagen, even though core fucosylation occurred (Fig. 4c). These results of conformational changes attributable to the bisecting N-glycan are consistent with the previous NMR analysis, which suggested that a bisecting GlcNAc in a complex type N-glycan not only restricted the conformational flexibility of the Manα1–6Man linkage, but also induced a uniquely constrained Manα1–3Man conformation by intra-glycan interactions [34]. In summary, with the core fucosylated GlcNAc covalently linked to SPARC protein, the biantennary arm of the Manα1–6Man linkage was observed in the molecular dynamics to bend toward the protein moiety of SPARC, thus incurring its collagen binding (Fig. 4a). In contrast, without core fucosylation at N116, both biantennary arms of the N-glycan were freely extended with full flexibility and that neither arm made contact with the SPARC protein nor collagen molecule to enable interactions. On the other hand, when a bisecting GlcNAc appeared in the N-glycan structure, the biantennary arm of the Manα1–6Man linkage was bend over in a way not to reach the collagen binding site and was unable to trigger collagen binding, even though the N-glycan was core fucosylated (Fig. 4c). Thus, our results suggest that core fucosylation may incur collagen binding by SPARC; however, this effect of core fucosylation was disabled when there is a unique bisecting GlcNAc in the N-glycan structure (Fig. 4c). Overall, our SPARC glycoprotein modelling results allude not only to a protein-protein interaction, but also to a glycan-protein complex, such that molecular movements in the complex influence SPARC-collagen binding; these movements in turn are affected by the presence of core fucosylation and its effects on the mobility of adjacent glycan structures. These molecular dynamics simulation results are also consistent with the findings with the Biacore binding assays described above. This approach with the combined use of Biacore binding assays and molecular dynamics simulations, thus, provide a useful tool for understanding the conformational effects of the glycan part of SPARC on its interaction with collagen.

### Role of Globo H ceramide in the promotion of angiogenesis

#### Glycosphingolipids

GSLs are a class of complex glycolipid expressed in all eukaryotic plasma membranes, and have diverse functions in normal development and cancer [18]. Aberrant expression of sugar is a unique feature of cancer cells [8, 18, 25, 29, 73]. In this rapidly advancing era of anticancer therapeutics, the targets for the current anticancer therapeutics are all directed against protein/glycoproteins on the cell surface of tumors. Recent FDA approval of anti-GD2 (a ganglioside) monoclonal antibody (dinutuximab) for neuroblastoma marked the first non-protein GSL target for anticancer therapy [72]. GHCer, which consists of a hexasaccharide, Fucα1-2Galβ1–3GalNAcβ1–3Galα1-4Galβ1-4Glcβ1, linked to ceramide, is the most prevalent cancer-associated (hyphen added) GSL. It is over-expressed in many epithelial cancers such as breast cancer and is being actively pursued as the next non-protein target for anticancer therapy [19]. However, the biological functions of GSLs, especially the glycan moiety, remain underexplored. In the following sections, we will highlight the features of molecular interactions between GSLs and their target proteins, emphasizing how conformational changes in the glycan moiety in GHCer could lead to alterations in protein interactions and resulting biological consequences. We will provide the scientific rationales for targeting GHCer for anticancer treatment and elucidate the approach for a rational design of GHCer-targeted therapeutics.

#### Role of GSL in proangiogenesis

Studies have reported in vitro and in vivo evidence supporting the proangiogenic activities of GHCer [9, 18, 73]. It was shown that GHCer was incorporated into human umbilical vein endothelial cells (HUVEC) through the uptake of microvesicles shed from Globo H-expressing tumor cells and that the addition of synthetic GHCer induced migration, tube formation, and intracellular Ca^2+^ mobilization [9]. In addition, the injection of GHCer-containing Matrigel plugs subcutaneously into mice induced greater formation of new blood vessels than plugs containing ceramide or PBS [9]. Furthermore, in cell sorting studies of breast cancer cells using two cell lines and one patient-derived xenograft, breast cancer cells expressing high levels of GHCer displayed greater tumor growth with higher blood vessel densities when compared with cells expressing low GHCer [9]. In clinical studies, GHCer-expressing breast cancer specimens contained higher vessel density than GHCer-negative specimens. Immunoprecipitation experiments revealed that translin-associated factor X (TRAX) was the predominant binding protein for GHCer [9]. Cellular studies have linked the angiogenic effects of GHCer to its co-localization with TRAX [9].

#### Prediction of GSL-protein interactions

##### GSL-binding site on TRAX

To delineate the molecular interactions between GSL and protein via in silico studies, the characteristics of the putative binding site in protein molecules need to be shown first. GSL consists of a hydrophobic ceramide part and a glycosidically bound carbohydrate part, which is hydrophilic. When contemplating the complex assembly of GSL and protein, such polarized features of GSL should be taken into consideration. In other words, a hydrophilic region in the targeted protein should be allocated for the carbohydrate head of GSLs, and a hydrophobic site allocated for the lipid tails. For example, X-ray crystallographic data for the complex between GSL and glycolipid transfer proteins [6] revealed that the Gal of GalCer interacted with hydrophilic AA of the transfer proteins via hydrogen bonds, whereas the ceramide part of GSL was lodged into a hydrophobic tunnel of the proteins [6]. Similarly, with molecular docking studies, we showed that TRAX interacted with GHCer in a region comprised of hydrophilic and hydrophobic areas [9].

##### The interactions between GHCer and TRAX

**Fig. 5:**
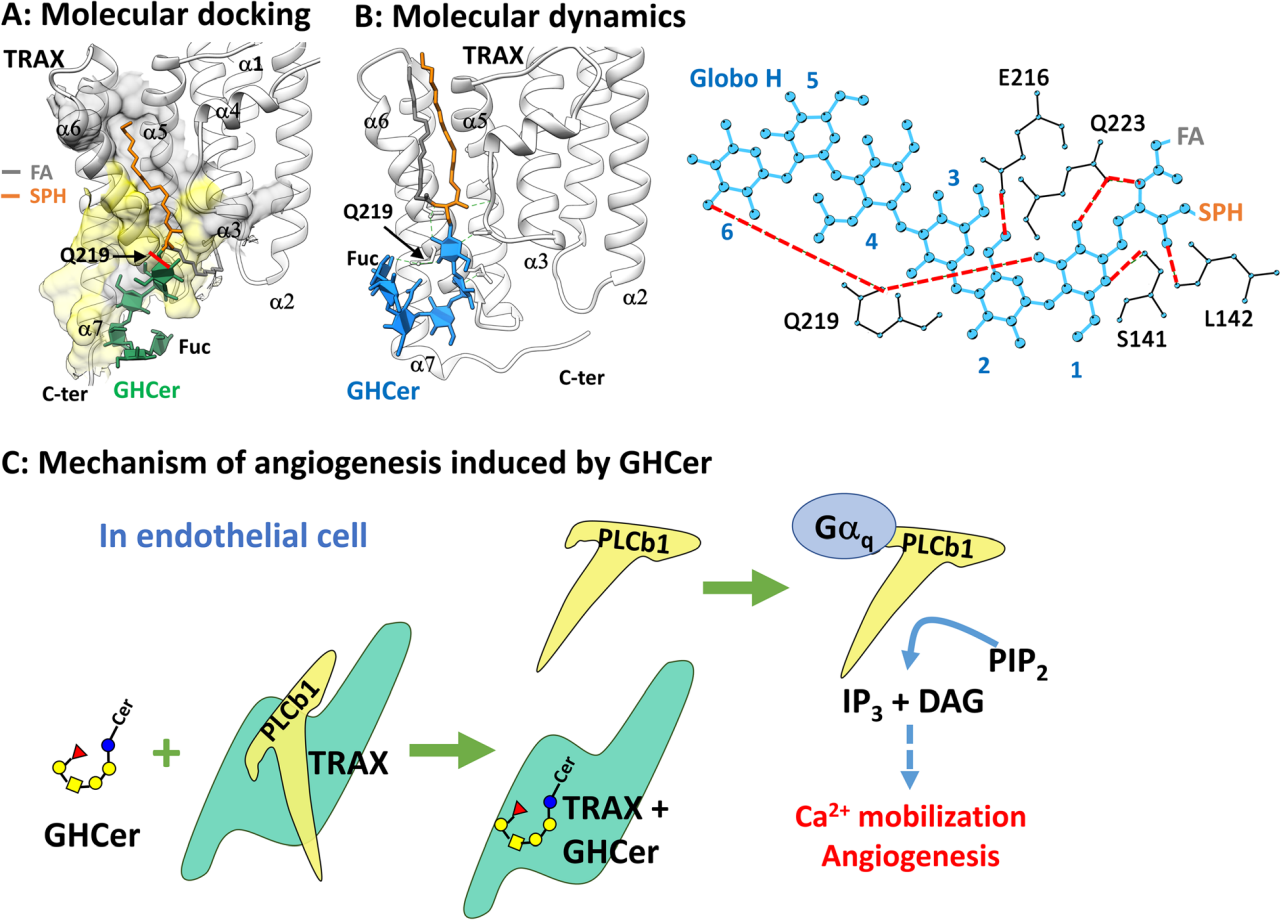
Mechanism of proangiogenic activity resulting from the binding of GHCer to TRAX. **a** Complex model of GHCer and TRAX predicted with molecular docking software in a previous study [9]; only one H-bond could be formed between GHCer and TRAX in this prediction. **b** GHCer-TRAX model predicted using the strategy of molecular dynamics simulation with a low-energy conformer of GHCer. GHCer formed as many as seven H-bonds interacting with E216, Q223, Q219, S141 and L142 on TRAX. **c** GHCer interacts with TRAX specifically, competing with PLCb1 and, thus, inducing calcium mobilization and angiogenesis in endothelial cells

As predicted with molecular docking in a previous study [9], Fig. 5a shows the complex structure between GHCer and TRAX. TRAX is an intracellular protein, which is composed of seven α helices (α1–α7) (PDB: 3PJA) [71]. The sphingosine chain (SPH) of GHCer was found to bind to a hydrophobic groove composed of α5 and α6 helices (gray surface in Fig. 5a) [9]; and the fatty acid chain (FA) bound to a cavity below α3 (Fig. 5a) [9]. In contrast, the glycan part of GHCer interacted with a hydrophilic area involving both the α6 and α7 helices near the C-terminal region of TRAX (yellow surface in Fig. 5a) [9]. It was noted that only one H-bond could be formed between GHCer and TRAX in this prediction [9], as shown at Q219 in Fig. 5a.

As mentioned, glycans usually bind to proteins in glycoconjugates in a low-energy state [35, 66] (Fig. 1c). Therefore, as illustrated in Fig. 1d–e, we described a unique strategy using low-energy glycan conformers for assembling glycan-protein complexes with molecular dynamics simulations. Here, we used a similar strategy to refine the molecular interactions within the GHCer-TRAX complex. The results were also compared with those from a previously reported model using conventional molecular docking [9].

First, we performed a 20-ns molecular dynamics simulation for Globo H (the sugar part of GHCer) in solution in the absence of TRAX, and found its low energy conformers, using Gromacs software. As shown in Fig. S1A, when reaching the equilibrium state, the resulting conformer of the 20-ns dynamics simulation revealed that Globo H displayed a U-shaped conformation, characterized by a unique configuration for the #6 fucose moiety. It was found that this fucose formed two intramolecular H-bonds with the #4 and #5 saccharide units of Globo H (red dashed lines in Fig. S1A). Subsequently, as described in Fig. 1e, this low-energy conformer of Globo H was then adopted for assembling with TRAX in a further molecular dynamics simulation, thereby generating a new predicted complex model as shown in Fig. 5b.

Compared to the findings illustrated in Fig. 5a, both the FA and SPH chains bound to the groove between α5 and α6 helices in the new model, as shown in Fig. 5b. In contrast to the molecular docking conformer (Fig. 5a), the U-shaped Globo H conformer predicted by molecular dynamics simulations crossed the α6 helix with the terminal fucose oriented in close proximity to the Q219 of TRAX (Fig. 5b). The close proximity of the fucose moiety to Q219 (Fig. 5b) was in contrast with the molecular docking conformer in which fucose was pointed in the opposite direction away from Q219 (Fig. 5a). Moreover, based on the model predicted by molecular dynamics simulations, analysis of molecular interactions showed that GHCer formed as many as seven H-bonds interacting with E216, Q223, Q219, S141 and L142 on TRAX (red dashed lines in Fig. 5b). In addition, the fucose unit contributed also to one H-bond with Q219 of TRAX in this new model (Fig. 5b), whereas it did not interact with AA residues of TRAX in the previous molecular docking model (Fig. 5a).

Since a larger number of molecular interactions were discovered by the method of molecular dynamics than molecular docking, the complex model thus predicted (Fig. 5b) might be more stable than the model in Fig. 5a. To further illustrate the conformational differences between low-energy conformers of Globo H predicted by molecular dynamics and the molecular docking conformer (Fig. 5aand b), angles of the glycosidic linkages connecting each saccharide unit of Globo H were analyzed. As indicated in Fig. S1B, the angle “psi” refers to the angle for glycosidic linkage connected to the hydroxyl group of the former saccharide, whereas the angle “phi” is the angle formed with the next saccharide. The data for the last few ns of molecular dynamics simulation at an equilibrium state, which were considered as low-energy conformers for Globo H (a total of 501 conformers), were used to display the distribution of glycosidic angles (blue curves in Fig. S1B,). On the other hand, the glycosidic angles calculated based on the molecular docking conformer are shown with green dashed lines. As shown, significant differences between the docking conformer and the low-energy conformers were observed in the psi angles for saccharides #1–#2 and saccharides #2–#3 (Fig. S1B). The other glycosidic angles of the docking conformers were similar to those of low-energy conformers by molecular dynamics. Since these two glycosidic angles of the model from molecular docking were far away from the low energy distributions predicted by molecular dynamics, the docking conformer (Fig. 5a) previously reported [9] might be an unfavorable conformation for binding with TRAX. Overall, these results suggest that using the strategy of molecular dynamics simulations with low-energy conformers of glycans, as described in Fig. 1d–e, would provide a method with improved prediction for glycan-protein interactions.

##### Mechanism of proangiogenic activity conducted by GHCer

It was found that the binding site of GHCer overlapped with that of phospholipase C β1 (PLCb1) on TRAX in molecular docking assays [9]. TRAX can capture PLCb1 in the cytosol, preventing activation of the phospholipase by Gq protein alpha subunit (Gαq) protein [1]. Therefore, competition between PLCb1 and GHCer for binding to TRAX forms the basis for a proposed link to the proangiogenic mechanism of GHCer. As illustrated in Fig. 5c, the terminal fucose leads GHCer to exhibit a U-shaped conformation fitting to a binding site on TRAX via H-bonding and hydrophobic interactions. Therefore, due to the structural recognition by TRAX, GHCer can thus function as an inhibitor of TRAX, resulting in the release of PLCb1 and its subsequent activation by Gαq protein. The activated PLCb1 then promotes the hydrolysis of PIP2 and formation of IP3 and DAG, thereby eventually inducing Ca^2+^ mobilization and angiogenesis in the tumor microenvironment [73].

GSLs were traditionally known as a type of biomolecule residing on plasma membrane. GHCer was the first GSL explicitly shown to serve as binding partner for an intracellular protein like TRAX. But, in addition to GHCer discussed in this review, recent studies have implied that GSLs might play roles outside the context of plasma membrane. For example, GM1 gangliosides were shown to interact with chromatin at the inner aspects of nuclear envelope in neuronal cells, affecting the activity of promoters for epigenetic activation [51]. GD3 gangliosides was also shown to interact with microtubules and was associated mitochondria to correlate with apoptotic stimulation of T cells [49]. In addition, mechanisms of translocation of membrane lipids to other membrane sites or intracellular binding proteins had been proposed. For example, lipid trafficking might occur intracellularly through vesicular trafficking or through membrane-membrane contact sites between organelles [31]. Alternatively, glycolipid transfer protein could bind GSL molecules through association to plasma membrane, thereby inducing the translocation of membrane GSLs to the binding site in protein [6]. On the other hand, small amount of GHCer might exist in cytosol in soluble micellar form due to its hydrophilic hexasaccharide moiety. When TRAX possessed good binding affinity for GHCer, making it possible to capture GHCer from membrane, trafficking vesicles, or those soluble GHCer molecules in cytosol. These mechanisms might explain how GSL molecule like GHCer can interact with intracellular protein, TRAX, thereby promoting angiogenesis in tumor microenvironment.

## Conclusion

Glycosylation is not only a structural modification for a biomolecule, but also a potential conductor that regulates its biological functions. As the example for glycoprotein discussed in this review, the glycan composition of SPARC protein alters both glycan-protein conformation and interaction, regulating its binding ability for collagen. This glycan-regulating mechanism might play a role in the pathology of lung diseases, such as COPD. For glycosphingolipids, aside from being a tumor antigen, GHCer acts as an angiogenic factor in the tumor microenvironment. The mechanistic evidence for the interaction between GHCer and TRAX illustrates the contribution of the fucose moiety the ability of GHCer to interact with intracellular protein and exert angiogenic activity. These findings not only provide sound scientific rationales for targeting Globo H, but also facilitate rational design of new glycan-targeted anticancer immunotherapy.

## Supplementary information

**Additional file 1: Fig. S1**. The docking conformer was different from the low-energy conformers. (A) Globo H conformer predicted by a 20-ns molecular dynamics simulation. (B) For the docking conformer (green line), the psi angle of saccharides #1-#2 was 291.6°, whereas the angle distribution was about 120° for the same position of low-energy conformers (blue line). Moreover, the psi angle of saccharides #2-#3 for the docking conformer was 84.2°; but for low-energy conformers, the angle distribution was about 240° at this position. The other glycosidic angles of the docking conformer were similar to that of low-energy conformers, as shown by molecular dynamics simulations.

## Data Availability

All data generated or analyzed during this study are included in this published article and references.

## Abbreviations

AA: amino acid(s)
COPD: chronic obstructive pulmonary disease
EC: C-terminal extracellular calcium-binding domain
ECM: extracellular matrix
EMT: epithelial mesenchymal transition
ESCs: embryonic stem cells
EthCD: electron-transfer/higher-energy collision dissociation
FDA: Food and Drug Administration
FS: follistatin-like domain
Fut: fucosyltransferase
GHCer: Globo H ceramide
GSL: glycosphingolipid
HUVEC: human umbilical vein endothelial cells
Kd: dissociation constant
KO: knockout
LC-MS/MS: liquid chromatography-tandem mass spectrometry
MS: mass spectrometry
PDB: Protein Data Bank
PLCb1: phospholipase C β1
RMSD: root-mean-square deviation
RU: response units
SPARC: recreted protein acidic and rich in cysteine
SPR: surface plasmon resonance
TRAX: translin-associated factor X
WT: wild type
