# Supplementary material for: Dissecting the conformation of glycans and their interactions with proteins
Source: J Biomed Sci. 2020 Sep 9;27:93. doi: 10.1186/s12929-020-00684-5 (PMC7487937; doi:10.1186/s12929-020-00684-5)
Supplement: Supplementary file 1 — Additional file 1: Fig. S1. The docking conformer was different from the low-energy conformers. (A) Globo H conformer predicted by a 20-ns molecular dynamics simulation. (B) For the docking conformer (green line), the psi angle of saccharides #1-#2 was 291.6°, whereas the angle distribution was about 120° for the same position of low-energy conformers (blue line). Moreover, the psi angle of saccharides #2-#3 for the docking conformer was 84.2°; but for low-energy conformers, the angle distribution was about 240° at this position. The other glycosidic angles of the docking conformer were similar to that of low-energy conformers, as shown by molecular dynamics simulations. [file 12929_2020_684_MOESM1_ESM.docx]

**Supplementary Material**

**Dissecting the Conformation of Glycans and Their Interactions with Proteins**

Sheng-Hung Wang ^1^, Tsai-Jung Wu ^1^, Chien-Wei Lee ^1^, John Yu ^1, 2, *^

^1^ Institute of Stem Cell and Translational Cancer Research, Chang Gung Memorial Hospital at Linkou, and Chang Gung University, Taoyuan 333, Taiwan

^2^ Institute of Cellular and Organismic Biology, Academia Sinica, Taipei, Taiwan

**Supplementary Figure**

**Fig. S1**


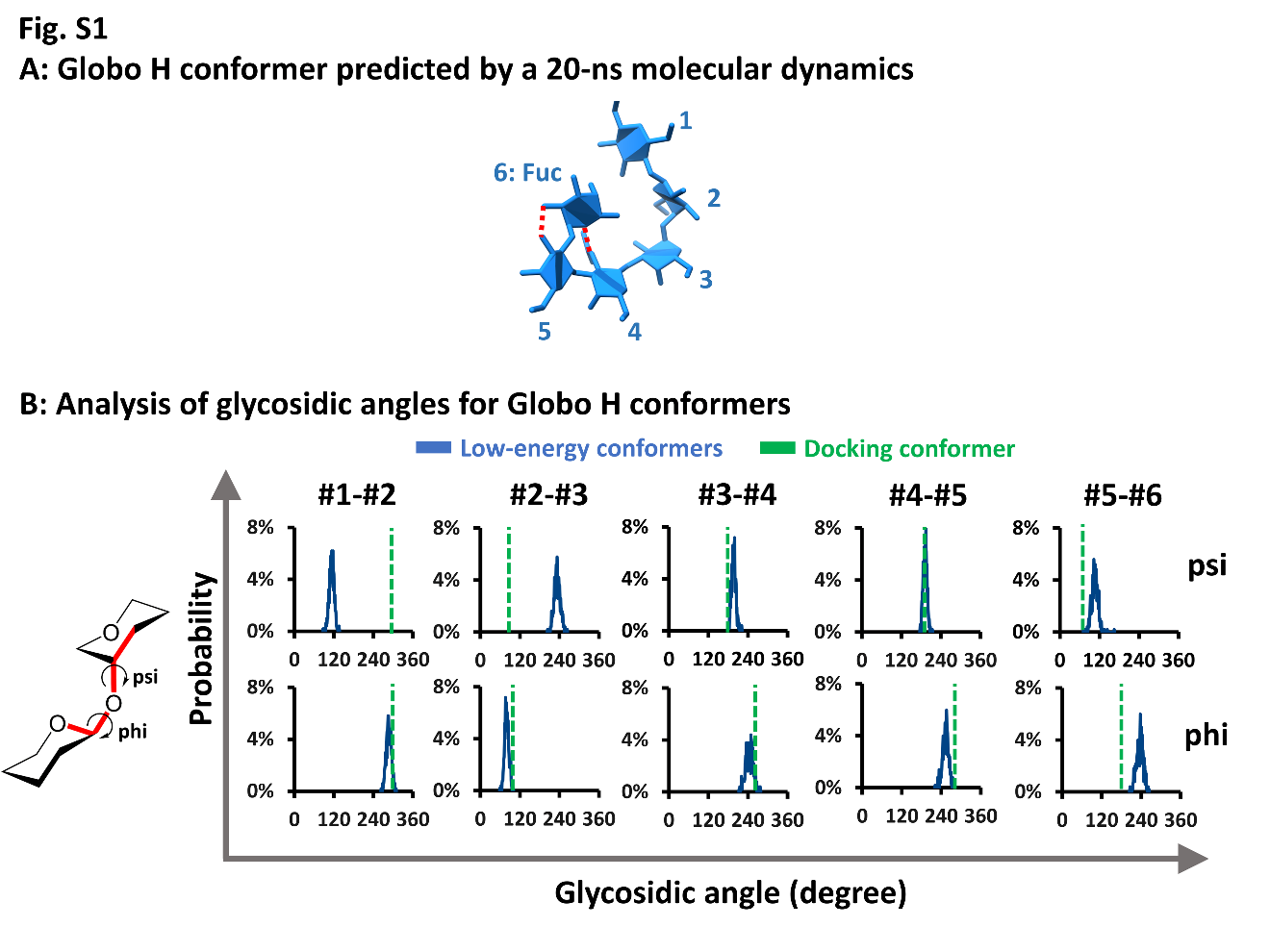


**Fig. S1. The docking conformer was different from the low-energy conformers**

(A) Globo H conformer predicted by a 20-ns molecular dynamics simulation.

(B) For the docking conformer (green line), the psi angle of saccharides #1-#2 was 291.6°, whereas the angle distribution was about 120° for the same position of low-energy conformers (blue line). Moreover, the psi angle of saccharides #2-#3 for the docking conformer was 84.2°; but for low-energy conformers, the angle distribution was about 240° at this position. The other glycosidic angles of the docking conformer were similar to that of low-energy conformers, as shown by molecular dynamics simulations.
